# Serum BDNF as an Objective Indicator of Rehabilitation Response in Patients with Subacute Stroke

**DOI:** 10.3390/jcm15145578

**Published:** 2026-07-16

**Authors:** Seyoung Shin, Heegoo Kim, Dae Hyun Kim, Won Hyuk Chang

**Affiliations:** 1 Department of Rehabilitation Medicine, CHA Bundang Medical Center, CHA University School of Medicine, Seongnam 13496, Republic of Korea; 2 Department of Physical and Rehabilitation Medicine, Center for Prevention and Rehabilitation, Heart Vascular Stroke Institute, Samsung Medical Center, Sungkyunkwan University School of Medicine, Seoul 06351, Republic of Korea; 3 Department of Health Science and Technology, Department of Medical Device Management and Research, Samsung Advanced Institute for Health Sciences and Technology (SAIHST), Sungkyunkwan University, Seoul 06351, Republic of Korea

**Keywords:** brain-derived neurotrophic factor, stroke rehabilitation, biomarker, subacute stroke, recovery of function

## Abstract

**Background/Objectives:** Serum brain-derived neurotrophic factor (BDNF) is linked to post-stroke neuroplasticity, but its temporal dynamics and clinical relevance during early rehabilitation remain unclear. We investigated changes in serum mature BDNF, proBDNF, and matrix metalloproteinase-9 (MMP-9) during the early subacute phase of stroke and their associations with functional improvement under standardized inpatient rehabilitation. **Methods:** In this prospective observational study at a tertiary inpatient rehabilitation unit, 100 stroke patients were enrolled and 84 were analyzed. Serum biomarkers and clinical assessments—including the National Institutes of Health Stroke Scale (NIHSS), Korean Mini-Mental State Examination, Fugl-Meyer Assessment, and Berg Balance Scale—were obtained at baseline (T0) and after two weeks of rehabilitation (T1). Correlation and multivariable regression analyses examined relationships between biomarker changes and functional outcomes, adjusting for age, sex, time from onset, and BDNF genotype. **Results:** An increase in serum mature BDNF from T0 to T1 was significantly associated with NIHSS improvement (r = −0.273, *p* = 0.014), which persisted after multivariable adjustment (β = −0.737, *p* = 0.009). Changes in proBDNF and MMP-9 showed no significant associations. **Conclusions:** Greater individual increases in serum mature BDNF during early subacute rehabilitation were independently associated with greater reductions in stroke severity, whereas the group-level mean did not increase over time. Serum mature BDNF may serve as a time-sensitive, objective biomarker of neurobiological recovery, potentially supporting individualized rehabilitation strategies.

## 1. Introduction

Stroke remains a leading cause of death and long-term disability worldwide, and its burden is projected to rise over the coming decades [1]. Neuroplasticity—the brain’s capacity to reorganize and form new neural connections after injury—underpins much of the functional improvement seen after stroke [2]. This plasticity involves complex processes such as synaptic remodeling, dendritic arborization, and axonal regeneration, and is modulated by molecular signaling cascades, genetic factors, and rehabilitation [3]. Given this multifactorial complexity, predicting recovery outcomes remains challenging, highlighting the need for reliable biomarkers that can reflect and guide successful brain repair processes. Up to now, the progress of functional recovery and the effectiveness of rehabilitation therapy in subacute stroke patients have been primarily evaluated through direct assessments of patient function.

Various assessment tools exist for motor, cognitive, and language functions, and these measures have been widely used and well validated [4,5]. Nevertheless, several limitations remain. First, functional assessments inherently require cooperation from patients with stroke. Most evaluations involve the patient’s ability to understand and execute specific instructions [6]. Consequently, their utility diminishes in patients with markedly impaired arousal or cognition, or in those admitted to intensive care units who cannot actively participate. Second, certain functional assessments can still suffer from inter-examiner variability. A representative example is the manual muscle test (MMT), where the distinction between grades 4 and 5 depends on whether the patient can resist the examiner’s applied force [7]. As such, the assessment may be influenced by the examiner’s and patient’s sex, age, and baseline muscle strength, introducing subjectivity. Therefore, it would be clinically meaningful to have an adjunctive marker that is not limited by patient cooperation and is free from examiner subjectivity, providing an objective measure of functional recovery and the effect of rehabilitation therapy.

One promising candidate for such a biomarker is serum brain-derived neurotrophic factor (BDNF). Among various neuroplasticity-related molecules, BDNF plays a central role in promoting neuronal survival, synaptic strengthening, and long-term potentiation [8]. Its precursor, proBDNF, exerts opposing effects by facilitating synaptic pruning and apoptosis. The enzymatic conversion of proBDNF to mature BDNF, mediated by molecules such as matrix metalloproteinase-9 (MMP-9), reflects a dynamic balance between neural repair and degeneration [9]. These biomolecular interactions are highly time-sensitive and may be influenced by the intensity and duration of rehabilitation therapy. A previous prospective study with patients with first-ever stroke showed that there were significant differences in BDNF level between those with low stroke severity and high stroke severity [10]. This study indicates that patients with a National Institutes of Health Stroke Scale (NIHSS) score below 6 on the first day of stroke had significantly higher BDNF levels compared to those with an NIHSS score above 6. Another study reported that serum BDNF level and stroke severity score had a negative correlation in stroke patients [11]. On the other hand, another study showed there was a significant negative non-linear cubic regression between BDNF concentration and stroke severity [12]. These discrepancies may partly reflect differences in the timing of sampling, single versus serial measurement, and assay specificity for total versus mature BDNF.

While previous studies have investigated the prognostic significance of serum BDNF levels after stroke, most relied on single-point measurements and did not account for temporal changes or ongoing neurorehabilitation. Serial sampling across the early subacute window, a period of heightened neuroplasticity, is therefore needed to capture the dynamic proBDNF-to-mature BDNF conversion that single-time-point designs cannot resolve. To address this limitation, the present study aims to examine whether changes in serum levels of mature BDNF, proBDNF, and MMP-9 during the early subacute phase could be associated with concurrent changes in clinical functional status, and to evaluate their potential as objective biomarkers that complement conventional clinical assessments of functional recovery and rehabilitation effect.

## 2. Materials and Methods

### 2.1. Study Design and Patients

The present analysis was conducted in the same prospective cohort described in our previous report [13], which found that baseline serum concentrations of mature BDNF, proBDNF, and MMP-9 had no independent prognostic value for functional recovery. The present study addresses a distinct question—whether changes in these biomarkers over the early subacute period are associated, at the individual level, with concurrent changes in clinical status—and reports non-overlapping analyses and results. This was a prospective, observational study conducted at a single hospital, involving patients admitted for stroke rehabilitation. Eligible participants met the following criteria: (1) diagnosis of unilateral stroke, (2) admission to the rehabilitation department within one month after stroke onset, and (3) presence of mild to severe motor deficits at the time of transfer to the rehabilitation department. Exclusion criteria included (1) progressive or medically unstable stroke, (2) a history of major neurological or psychiatric disorders, (3) significant systemic diseases affecting the liver, kidney, heart, or lungs, (4) terminal illness with an expected survival of less than one year, and (5) current pregnancy or lactation.

Patients were evaluated at two predefined time points: at entry to the rehabilitation unit following completion of acute stroke treatment (T0) and after two weeks of intensive inpatient rehabilitation (T1). The inpatient comprehensive rehabilitation program between T0 and T1 included two hours of physical therapy and one hour of occupational therapy daily on weekdays in accordance with the Clinical Practice Guidelines for Stroke Rehabilitation in Korea [14].

### 2.2. Data Acquisition and Biomarker Handling

Baseline demographic (age, sex, smoking and drinking history) and clinical information (stroke lateralization, etiology, onset timing, comorbidities, BMI, and BDNF genotype) were collected at T0. BDNF genotyping was performed at baseline. Whole blood was collected into EDTA tubes, followed by genomic DNA extraction from leukocytes using a standard protocol involving proteinase K and RNase digestion with subsequent phenol-chloroform purification. The BDNF Val66Met (rs6265) polymorphism was analyzed using PCR-restriction fragment length polymorphism methods [15]. Genotypes were categorized as Val/Val, Val/Met, or Met/Met and numerically coded based on the number of Met alleles (0, 1, or 2) for statistical modeling. Functional outcomes were assessed at each time point using standardized tools: stroke severity was evaluated via the NIH Stroke Scale (NIHSS) [16], cognitive function via the Korean version of the Mini-Mental State Examination (K-MMSE) [17], upper extremity motor function using the Fugl-Meyer Assessment (FMA) upper scores [18], balance with the Berg Balance Scale (BBS) [19], and depressive symptoms through the short form of the Geriatric Depression Scale (GDS) [20]. Trained and certified occupational and physical therapists conducted all assessments in person.

**Figure 1:**
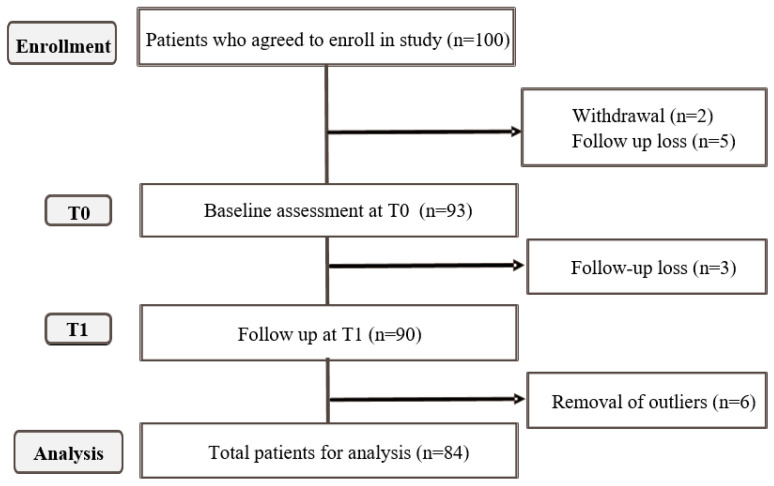
Flowchart illustrating the enrollment, follow-up, and inclusion of participants in the final analysis.

Venous blood samples were obtained at all time points (T0, T1), and Serum levels of mature BDNF, proBDNF, and MMP-9 were measured using the human BDNF ELISA Kit (Adipo Bioscience, Santa Clara, CA, USA), the human proBDNF ELISA Kit (Adipo Bioscience, Santa Clara, CA, USA), and the human MMP-9 ELISA Kit (R&D Systems, Minneapolis, MN, USA), respectively. The optical density of each well was measured using an automated microplate reader (Emax; Molecular Devices, Sunnyvale, CA, USA). To ensure data quality, outliers were identified and excluded using the interquartile range (IQR) method, defined as values falling below Q1 − 1.5 × IQR or above Q3 + 1.5 × IQR [21]. The numbers of the excluded values are presented in Figure 1. An intention-to-treat principle was applied, including all enrolled patients in the analyses regardless of dropouts or incomplete data.

### 2.3. Statistical Analysis

Baseline characteristics, functional assessments, and biomarker levels were summarized using descriptive statistics. Continuous variables were presented as means ± standard deviations or medians with interquartile ranges, depending on their distribution. Categorical variables were reported as frequencies and percentages.

To investigate the correlations between serum biomarker levels and patients’ characteristics and functional changes, Pearson correlation tests were conducted. The correlations between serum biomarker levels at T0 and T1, and the changes in functional status during the first two weeks of comprehensive inpatient rehabilitation (T0 to T1) were analyzed. Variables with a *p*-value less than 0.2 in correlation analysis were further examined using univariate linear regression analysis to indirectly explore potential causal relationships. A liberal screening threshold (*p* < 0.2) was applied to avoid prematurely excluding potential confounders, in line with recommendations for purposeful variable selection [22,23]; statistical significance for the final models was set at *p* < 0.05. After univariate linear regression, multivariable linear regression analyses were also performed to evaluate whether changes in serum biomarker levels during early rehabilitation were independently associated with changes in functional assessments after adjustments. To adjust for potential confounding factors, the following covariates were included in the model: age, sex, duration from stroke onset to T0, and BDNF genotype (Val66Met polymorphism). These covariates were selected based on their known influence on neuroplasticity, stroke recovery, or serum BDNF expression [24].

All statistical analyses were performed using R (version 4.0.3; R Foundation for Statistical Computing, Vienna, Austria).

## 3. Results

Of the 100 participants initially enrolled in the study, 93 patients completed the baseline assessment at T0 (with 2 participants withdrawing and 5 lost to follow-up). For the serum BDNF analysis at T1, only those with available T1 BDNF data were included. After excluding an additional 3 participants due to follow-up loss and 6 identified as outliers, a total of 84 patients were included in the final analysis (Figure 1). Table 1 summarizes the clinicodemographic characteristics, including vascular risk factors, stroke subtype, comorbidities, and baseline serum biomarker levels. The mean age of the patients was 63.5 ± 14.4 years, and 47 patients (56.0%) were male. Fifty-nine patients with stroke (70.2%) were ischemic stroke, and the majority had hypertension (*n* = 59, 71.1%). The mean time from stroke onset to transfer to the rehabilitation department (T0) was 16.2 ± 7.9 days. The overall stroke severity at T0 was in moderate severity range (NIHSS 7.0 ± 4.8). The distribution of BDNF genotypes was as follows: Val/Val (25.0%), Val/Met (47.6%), and Met/Met (27.4%).

**Figure 2:**
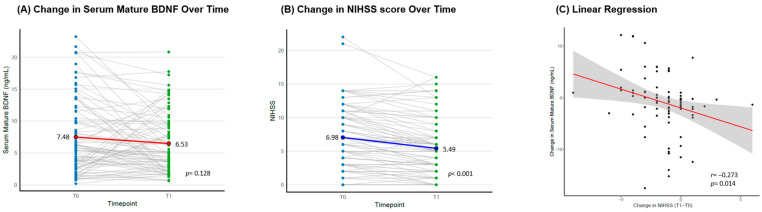
Changes in serum mature BDNF and stroke severity during early rehabilitation and their association. (**A**) Individual and group-mean trajectories of serum mature BDNF (ng/mL) between T0 (upon transfer to the rehabilitation department) and T1 (2 weeks after transfer). Gray lines indicate individual patients, and the red line represents the group mean (±SD). (**B**) Changes in NIHSS scores over the same period; gray lines show individual patients and the blue line represents the group mean (±SD). (**C**) Scatter plot with linear regression line (red) and 95% confidence interval (gray shading) showing an inverse relationship between the change in NIHSS (T1–T0) and the change in serum mature BDNF (*r* = −0.273, *p* = 0.014, *n* = 81); greater individual increases in serum mature BDNF were associated with greater reductions in stroke severity.

At T0, the mean serum mature BDNF level was 7.5 ± 5.9 ng/mL, and the MMP-9 level was 348.6 ± 185.5 ng/mL. The changes in individual NIHSS score and mature BDNF level are demonstrated in Figure 2A,B.

### 3.1. Correlation Analysis Between Serum Biomarker Changes and Functional Outcomes

To explore whether short-term changes in serum biomarkers were associated with early functional improvements during the subacute phase, Pearson’s correlation analyses were conducted between the change in serum biomarker levels and the corresponding changes in clinical outcomes from T0 to T1 (ΔT1T0) (Table 2).

A significant negative correlation was observed between the change in mature BDNF levels and the improvement in stroke severity, as measured by the NIHSS score (*r* = −0.273, *p* = 0.014), suggesting that an increase in mature BDNF during early rehabilitation was associated with a greater reduction in stroke severity. No significant correlations were found between mature BDNF changes and other functional assessments. At the group level, mean serum mature BDNF did not change significantly from T0 to T1 (7.48 vs. 6.53 ng/mL; paired *p* = 0.13).

For proBDNF, there tended to be a weak negative correlation with the change in cognitive function (K-MMSE) from T0 to T1 without statistical significance (*r* = −0.181, *p* = 0.145). Changes in MMP-9 levels were not significantly correlated with any functional assessments.

### 3.2. Univariate and Multivariable Linear Regression Analysis

Variables that showed potential associations in the correlation analysis (*p* < 0.2) were further examined using univariate linear regression (Table 3). The change in mature BDNF (ΔT1T0) remained significantly associated with improvement in NIHSS score (*β* = −0.729, *p* = 0.013), confirming the negative association between increasing BDNF and decreasing stroke severity (Figure 2C). In contrast, the change in proBDNF was not significantly associated with any of the functional outcomes in regression analysis.

Table 4 shows the multivariable linear regression analysis with adjustment factors (age, sex, stroke onset to T0 days, BDNF genotype) examining the association between changes in serum mature BDNF levels and changes in NIHSS scores during the early subacute phase. A greater improvement in stroke severity (ΔNIHSS T1T0) was independently associated with a greater increase in serum BDNF levels (*β* = −0.737, *p* = 0.009). Male patients showed significantly higher BDNF changes compared to female patients (*β* = −4.319, *p* = 0.001). Age was marginally associated with BDNF changes (*β* = 0.068, *p* = 0.096), while neither the duration from stroke onset to T0 nor BDNF genotype showed significant associations. These findings suggest that changes in serum BDNF are independently associated with early functional recovery after stroke, after accounting for age, sex, and genetic factors.

## 4. Discussion

This study explored the changes in serum mature BDNF, proBDNF, and MMP-9 during the subacute phase of stroke, with particular attention to their dynamic association with functional recovery under comprehensive rehabilitation. Our findings highlight that changes in serum mature BDNF levels were significantly associated with improvement in stroke severity, as measured by NIHSS scores, over a two-week rehabilitation period. This association remained robust even after adjusting for key demographic and clinical covariates, including age, sex, time from onset, and BDNF genotype. Notably, this pattern was not observed for proBDNF or MMP-9, suggesting that among the three potential serum markers examined, mature BDNF can be the most responsive to short-term rehabilitation-mediated neural recovery.

In this study, we conducted a comprehensive analysis of the relationship between serial changes in serum BDNF levels and stroke severity in subacute stroke patients who were receiving standardized and consistent inpatient rehabilitation. NIHSS measured for stroke severity is a standardized and objective clinical tool used to quantify the neurological deficits of a patient with stroke. The scale evaluates multiple domains of neurological function, including motor strength, language, level of consciousness, visual fields, and sensation. By providing a composite score based on the patient’s performance, the NIHSS serves as a reliable measure of their current neurological status and the overall severity of the stroke-related impairment [25]. By evaluating both biomarkers and functional status at two time points, we were able to capture the temporal dynamics of neuroplasticity-related changes during a clinically relevant recovery window. The study employed a multi-layered statistical approach—including correlation analysis, univariate regression, and multivariable regression with key covariates such as age, sex, time from onset, and BDNF genotype—to explore the association between changes in serum BDNF and functional improvement. This design allowed for a more robust assessment of whether BDNF dynamics serve as an independent indicator of stroke recovery. The controlled rehabilitation environment minimized external variability, enhancing the internal validity of the findings. Moreover, by focusing on change values rather than absolute levels, the study addressed inter-individual variability in baseline BDNF concentrations, offering a more individualized and clinically meaningful perspective.

Many previous studies showed that elevated BDNF levels support neuroplasticity and are associated with better recovery trajectories in stroke patients, measuring serum often at baseline with or without a single follow-up time point [24,26,27]. Compared with these studies, this study adopted a shorter follow-up interval of two weeks and focused on the early subacute phase, a period considered most favorable for neuroplasticity. As a result, this study demonstrated that during the inpatient rehabilitation period, increases in serum BDNF levels were inversely associated with stroke severity, suggesting a potential causal link. Together, these findings indicate that changes in serum BDNF levels may serve as a promising biomarker reflecting the effectiveness of stroke rehabilitation and the extent of functional recovery. Kazakov S. et al. [28] reported that BDNF levels increased in patients who received rehabilitation during the early subacute phase, whereas those discharged without rehabilitation showed a decrease in BDNF concentrations. These findings are consistent with the present study, which lends support to the hypothesis that participation in rehabilitation and physical activity regulate serum BDNF levels.

A recent randomized controlled trial reported that serum BDNF increased by approximately 18% following an 8-week aerobic exercise program in subacute stroke survivors, with no change in the usual-care control group [29]. Conversely, another recent trial found no significant change in serum mature BDNF despite marked improvements in cardiorespiratory fitness [30], a discrepancy likely attributable to differences in the BDNF fraction measured, cohort severity, and exercise intensity. Together, these findings suggest that serum BDNF may reflect rehabilitation-related neuroplasticity, while also indicating that such dynamics may not be consistently captured at the group-mean level—consistent with our observation that individual, rather than mean, changes in mature BDNF were associated with functional improvement.

In patients with Parkinson’s disease, serum BDNF has been used as a biomarker to reflect rehabilitation efficacy [31,32]. A meta-analysis further demonstrated that such increases in BDNF were associated with improvements in clinical outcomes, including motor symptoms measured by the Unified Parkinson’s Disease Rating Scale and other assessments, suggesting that BDNF may serve as a reliable indicator of the effectiveness of rehabilitation interventions [33]. Based on our findings, serum BDNF levels may, as in Parkinson’s disease, serve as an objective marker in stroke patients, complementing conventional functional assessments by reflecting the efficacy of rehabilitation and the extent of recovery.

### Study Limitations

Nevertheless, some limitations warrant consideration. The sample size, while adequate for initial modeling, may have constrained the detection of subtler associations. This study’s inclusion criteria were limited to patients with motor dysfunction, particularly those with hemiparetic stroke. Consequently, the generalizability of the findings to all stroke patients could be limited. Additionally, the exclusion of outliers via the IQR method may have inadvertently removed meaningful extremes, particularly for low-concentration markers like proBDNF. Finally, changes in serum BDNF were significantly associated only with NIHSS scores, whereas no clear associations were observed with individual functional domains such as motor, balance, or cognition. This may be explained by the fact that NIHSS provides an integrated measure encompassing multiple functional aspects [16], while our data included patients with heterogeneous patterns of impairment across domains. Future studies should further explore the relationship between BDNF dynamics and specific functional domains using a larger number of participants.

## 5. Conclusions

This study suggests that serum mature BDNF holds promise as a time-sensitive biomarker reflecting neurobiological recovery during stroke rehabilitation in patients with early subacute stroke. Future research involving larger and more diverse populations should aim to validate these findings and further explore the mechanistic underpinnings of BDNF responsiveness.

## Figures and Tables

**Table 1:** Clinicodemographic patient characteristics (*n* = 84).

Characteristic	Value
Age, yr	63.5 ± 14.4
Sex (male:female)	47 (56.0%):37 (44.0%)
Stroke type (ischemic:hemorrhagic)	59 (70.2%):25 (29.8%)
Duration from stroke onset to T0, d	16.2 ± 7.9
BDNF genotype (Val/Val:Val/Met:Met/Met)	21 (25.0%):40 (47.6%):23 (27.4%)
Smoking	13 (15.7%)
Alcohol	34 (41.0%)
**Comorbidities**	
Hypertension	59 (71.1%)
Diabetes mellitus	32 (38.6%)
Dementia	2 (2.4%)
Depression	3 (3.6%)
**Baseline serum biomarker levels**	
Mature BDNF, ng/mL	7.5 ± 5.9
ProBDNF, ng/mL	0.5 ± 0.6
MMP-9, ng/mL	348.6 ± 185.5
**Baseline stroke severity and functional status**	
NIHSS	7.0 ± 4.8
FMA	21.6 ± 19.7
BBS	12.2 ± 14.5
K-MMSE	19.3 ± 10.9
GDS	7.9 ± 4.1

Continuous variables are expressed as mean ± standard deviation, while categorical variables are presented as *n* (%). Abbreviations: BDNF, brain-derived neurotrophic factor; MMP-9, matrix metalloproteinase-9; NIHSS, National Institutes of Health Stroke Scale; K-MMSE, Korean Mini-Mental State Examination; FMA, Fugl-Meyer Assessment; BBS, Berg Balance Scale; GDS, Geriatric Depression Scale.

**Table 2:** Pearson’s correlation analysis between changes in serum biomarker level and changes in functional assessments during the subacute phase.

Variables	Mature BDNF ΔT1T0		ProBDNF ΔT1T0		MMP-9 ΔT1T0	
	*r*	*p*	*r*	*p*	*r*	*p*
NIHSS ΔT1T0	−0.273	0.014 ^#^	−0.030	0.809	0.017	0.886
FMA upper ΔT1T0	−0.002	0.988	0.026	0.833	0.060	0.609
BBS ΔT1T0	−0.081	0.588	0.075	0.657	−0.020	0.903
K-MMSE ΔT1T0	0.094	0.401	−0.181	0.145 ^#^	−0.073	0.533
GDS ΔT1T0	−0.0003	0.998	0.070	0.606	0.028	0.825

Estimated coefficient *r* and *p* values from Pearson’s correlation analysis. ^#^ *p* < 0.2. Abbreviations: BDNF, brain-derived neurotrophic factor; MMP-9, matrix metalloproteinase-9; T0, completion of acute stroke care; T1, 2 weeks after transfer to the rehabilitation department; NIHSS, National Institutes of Health Stroke Scale; K-MMSE, Korean Mini-Mental State Examination; FMA, Fugl-Meyer Assessment; BBS, Berg Balance Scale; GDS, Geriatric Depression Scale.

**Table 3:** Univariate linear regression analysis between changes in serum biomarker level and changes in functional assessments during the subacute phase.

*Y*	*X*	Estimate *β* (95% CI)	SE	*t*	*p*
Mature BDNF ΔT1T0	NIHSS ΔT1T0	−0.729 (−1.30, −0.15)	0.289	−2.521	0.013
ProBDNF ΔT1T0	K-MMSE ΔT1T0	0.112 (−0.15, 0.38)	0.132	0.845	0.401

Estimated coefficient *β*, *t*-value and *p* values from univariate linear regression analysis. Values are from separate univariate models: *n* = 81 (mature BDNF–NIHSS; R^2^ = 0.074) and *n* = 71 (proBDNF–K-MMSE; R^2^ = 0.031). β denotes the change in serum biomarker (ng/mL) per 1-point change in the clinical score; CI, confidence interval. *p* < 0.05. Abbreviations: BDNF, brain-derived neurotrophic factor; NIHSS, National Institutes of Health Stroke Scale; K-MMSE, Korean Mini-Mental State Examination; T0, completion of acute stroke care; T1, 2 weeks after transfer to the rehabilitation department.

**Table 4:** Multivariable linear regression analysis between changes in serum BDNF level and changes in NIHSS during the subacute phase.

*Y*	*X*	Estimate *β* (95% CI)	SE	*t*	*p*
Mature BDNF ΔT1T0	(Intercept)	1.363 (−5.78, 8.51)	3.587	0.380	0.705
	NIHSS ΔT1T0	−0.737 (−1.28, −0.19)	0.274	−2.689	0.009
	Age	0.068 (−0.01, 0.15)	0.040	1.687	0.096
	Sex (Ref: Male)	−4.319 (−6.71, −1.93)	1.199	−3.602	0.001
	Onset to T0 days	0.046 (−0.10, 0.19)	0.074	0.623	0.535
	BDNF genotype, Met/Met (Ref: Val carriers)	−1.131 (−3.83, 1.57)	1.354	−0.835	0.406

Estimated coefficient *β*, *t*-value and *p* values from multivariable linear regression analysis with covariates (age, sex, onset to T0 days, BDNF genotype). The model included 81 patients (R^2^ = 0.228, adjusted R^2^ = 0.176; F(5, 75) = 4.42, *p* = 0.001). β denotes the change in serum mature BDNF (ng/mL) per unit of each covariate (per 1-point NIHSS change, per year of age, female vs. male, per day from onset, and Met/Met vs. Val carriers); CI, confidence interval. *p* < 0.05. Abbreviations: BDNF, brain-derived neurotrophic factor; NIHSS, National Institutes of Health Stroke Scale; T0, completion of acute stroke care; T1, 2 weeks after transfer to the rehabilitation department.

## Data Availability

De-identified patient data, the study protocol, and results will be shared upon reasonable request to the corresponding author. Data sharing with researchers who provide a methodologically sound proposal will be available beginning 3 months and ending 60 months after article publication. Shared data may only be used to achieve the aims of approved proposals. Proposals should be directed to wh.chang@samsung.com; to gain access, data requestors must sign a data access agreement.
